# Factors Associated with Sarcopenia and 7-Year Mortality in Very Old Patients with Hip Fracture Admitted to Rehabilitation Units: A Pragmatic Study

**DOI:** 10.3390/nu11092243

**Published:** 2019-09-18

**Authors:** Vincenzo Malafarina, Concetta Malafarina, Arantzazu Biain Ugarte, J. Alfredo Martinez, Itziar Abete Goñi, M. Angeles Zulet

**Affiliations:** 1 Department of Nutrition, Food Science and Physiology, School of Pharmacy and Nutrition, Universidad de Navarra, 31008 Pamplona, Spain; jalfmtz@unav.es (J.A.M.); iabetego@unav.es (I.A.G.); mazulet@unav.es (M.A.Z.); 2 Department of Geriatrics, Complejo Hospitalario de Navarra, Calle de Irunlarrea 3, 31008 Pamplona, Spain; 3 Department of Radiology, Complejo Hospitalario de Navarra, 31008 Pamplona, Spain; concetta.malafarina@navarra.es; 4 Nursing Department, Hospitalización a Domicilio, Complejo Hospitalario de Navarra, 31008 Pamplona, Spain; abiauga@alumni.unav.es; 5 CIBERobn, Physiopathology of Obesity and Nutrition, Instituto de Salud Carlos III, 28029 Madrid, Spain; 6 Centre for Nutrition Research, School of Pharmacy and Nutrition, Universidad de Navarra, 31008 Pamplona, Spain; 7 Navarra Institute for Health Research (IdiSNA), 31008 Pamplona, Spain

**Keywords:** skeletal muscle mass, sarcopenia, hip fracture, rehabilitation unit, mortality, very old patients, undernutrition

## Abstract

Background: Admitted bedridden older patients are at risk of the development of sarcopenia during hospital stay (incident sarcopenia). The objective of this study was to assess the factors associated with sarcopenia (incident and chronic) and its impact on mortality in older people with hip fracture. Methods: A multicenter, pragmatic, prospective observational study was designed. Older subjects with hip fracture admitted to two rehabilitation units were included. Sarcopenia was assessed at admission and at discharge according to the revised EWGSOP (European Working Group on Sarcopenia in Older People) consensus definition. The mortality was evaluated after 7 years of follow-up. Results: A total of 187 subjects (73.8% women) age 85.2 ± 6.3 years were included. Risk factors associated to incident and chronic sarcopenia were undernutrition (body mass index—BMI and Mini Nutritional Assessment−Short Form—MNA-SF), hand-grip strength and skeletal muscle index. During follow-up 114 patients died (60.5% sarcopenic vs. 39.5% non-sarcopenic, *p* = 0.001). Cox regression analyses showed that factors associated to increased risk of mortality were sarcopenia (HR: 1.67, 95% CI 1.11–2.51) and low hand-grip strength (HR: 1.76, 95% CI 1.08–2.88). Conclusions: Older patients with undernutrition have a higher risk of developing sarcopenia during hospital stay, and sarcopenic patients have almost two times more risk of mortality than non-sarcopenic patients during follow-up after hip fracture.

## 1. Introduction

Sarcopenia is a geriatric syndrome [1], characterized by the loss of muscle mass and function, which has been recognized as an independent clinical syndrome by the International Classification of Disease, tenth revision, Clinical Modification (ICD-10CM) code (M62.84), thus strengthening its clinical significance [2,3]. In 2019 the European Working Group on Sarcopenia in Older People (EWGSOP) published the revised criteria for sarcopenia diagnosis, which included low hand-grip strength, low muscle mass and low gait speed [4].

Sarcopenia shares physio-pathological mechanisms, and is associated with a high prevalence of osteoporosis [5,6]. This situation has brought the introduction of the concept of osteosarcopenia, where sarcopenia and osteoporosis overlap resulting in important functional consequences like falls and hip fractures among others [7,8].

Hip fractures have an important impact upon the daily lives of people above 65 years, as over 40% of those who suffer a hip fracture do not recover the functional status they had prior to the fracture [9], and sarcopenia could be a factor influencing the loss of function [10]. In addition, hip fractures are associated with a high prevalence of institutionalization and mortality after 3 years (above 35%) [11]. Malnutrition is one of the physio-pathological mechanisms involved in the development of sarcopenia [12,13,14]. Oral supplementation has shown to be an important treatment for muscle mass preservation in older people with hip fracture [15,16].

The prevalence of sarcopenia in older people admitted to acute units with hip fractures varies between 17% and 37% [17,18], and increases in rehabilitation units up to 34% and 59% [19,20]. However, the percentage of very old subjects included in studies carried out in rehabilitation units usually is very low [19]. Therefore, the mean age of subjects included is not representative of older patients with hip fracture.

Sarcopenia is an important prognostic factor for mortality in older patients admitted to acute units [21,22,23,24]. Evidence regarding the effect of sarcopenia on the survival of older people with a hip fracture admitted to rehabilitation units is relatively limited. 

Older people are often excluded from clinical trials [25], thus results of such trials have limited relevance to clinical practice [26]. In this sense, results of pragmatic studies enrolling real-life older people are very important to help in the daily clinical practice [27,28].

In this sense, the aim of this pragmatic study was to identify relevant factors associated to the presence of sarcopenia at admission and to the development of sarcopenia during a hospital stay in older patients admitted to post-acute wards for hip fracture rehabilitation, and to investigate the influence of sarcopenia on the risk of mortality after 7 years of follow-up.

## 2. Methods

### 2.1. Study Population

This pragmatic, prospective observational study included 187 subjects, older than 65 years of age admitted for rehabilitation after surgery due to hip fracture. The study was carried out at the post-acute rehabilitation units of two hospitals: Hospital San Juan de Dios in Pamplona (between January 2012 and August 2014), and Hospital Viamed Valvanera in Logroño (between November 2014 and December 2015), both located in Spain. The characteristics of these rehabilitation units have already been described [15]. Briefly, all those subjects suffering from a hip fracture are subjected to an orthopedic surgery. Patients with active medical processes who cannot return to their homes are referred to rehabilitation units, which are physically separate from hospitals for acute cases. Patients with pathological or periprotesic fractures, patients with fractures caused by traffic accidents, those with active oncologic pathologies and those receiving palliative care with a life expectancy of less than one year were not included in the study.

The protocol for this study was approved by the local ethics committee (Comité de Ética de Investigación Clínica de la Comunidad Foral de Navarra; Code number No. 33 of 17/2/2012). All subjects included signed informed consent forms. The protocol for this study was registered at the NIH ClinicalTrials.gov on November 22, 2011 (Identifier: NCT01477086). The study was carried out in accordance with the Helsinki Declaration. 

### 2.2. Sarcopenia Assessment 

Sarcopenia assessment was carried out upon admission, according to revised EWGSOP consensus definition [4]. Grip strength was measured with a digital dynamometer (DynX^®^ Akern, Florence) following the standard protocol [29]. Grip strength was measured in both hands, and the better of two trials with each hand was recorded. Scores <27 kg in men or <16 kg in women were considered as low grip strength [4]. Muscle mass was assessed by bioimpedance analysis (BIA, Akern, Florence), resulting in scores for resistance (Rz) and reactance (Xc) expressed in ohms. The phase angle (PA) was also calculated, and expressed in degrees. The phase angle could be a representative marker of cell membrane integrity and vitality [30]. Low PA was considered for values <4.5 degrees [31]. Measurements were made in the morning, on fasting state, placing the electrodes (BIA Akern electrodes) on the ankle of the leg not subjected to surgery and on the wrist on the same side. Patients were placed in a supine position, on a non-conductive surface, arms separated from the upper body and legs slightly apart, so as to avoid contact between them. Appendicular skeletal muscle mass (ASMM) was calculated according to the formula [32]:

ASMM = −3.964 + (0.227 × RI) + (0.095 × weight) + (1.384 × sex) + (0.064 × Xc)(1)

where RI is the resistive index calculated as (height in centimeters squared/Rz), weight in kg, and for the sex variable assuming values of 0 for women and 1 for men. Among the formulae validated for the assessment of muscle mass with BIA in older people, the formula put forward by Sergi et al. has demonstrated higher sensitivity and specificity [33]. The skeletal muscle mass index (SMI), corrected for height, was calculated with the following formula: SMI = ASMM/height in meters squared. SMI was defined as reduced for scores <7.0 kg/m^2^ in men and <6.0 kg/m^2^ in women [4]. 

Walking speed was measured with the 4 m walking test, considering scores ≤0.8 m/s as low gait speed. This measurement has been used as criterion for the sarcopenia severity assessment. Both measurements, muscle mass by BIA and hand-grip strength were carried out upon admission and 48 h before discharge. Walking speed was only measured upon discharge, due to patients’ inability to walk steadily upon admission.

Sarcopenic patients have been defined as those with low hand-grip strength and low SMI considering the EWGSOP criteria recently published [4].

### 2.3. Patients Assessment

Demographic data (age, sex and marital status) were collected through an interview with the patient or a direct relative. Data concerning the type of fracture, time to surgery, type of surgery as well as post-surgery complications were collected throughout the medical history. The assessment of the nutritional status as well as anthropometric measurements (weight and height) and sarcopenia assessment (hand-grip strength, bio-impedance analysis and gait speed) were carried out by direct examination. Nutritional assessment was carried out by a nutritionist who used the Mini Nutritional Assessment-Short Form (MNA-SF) that classified patients as well nourished (12–14 points), at risk of malnutrition (8–11 points) and malnourished (0–7 points) [34]. Changes of weight, grip strength and SMI have been calculated as the difference between discharge and admission.

Functional capacity in the activities of daily living (ADL) was assessed by means of the Barthel index [35], which scores range from 0, totally dependent and 100, totally independent in the ADL. Information on previous BI scores from 15 days before the fracture was collected by an interview with patients or caregivers and upon discharge by direct examination (24 h before discharge). Cognitive capacities were assessed with the Spanish version of the Mini Mental State Examination (MMSE) [36]. MMSE was carried out during the first two weeks after admission to hospital.

Blood samples were analyzed in the first 72 h since admission and 48 h prior to discharge in order to determine hemoglobin, total proteins and albumin, Vitamin-D concentration and kidney function. In addition, inflammatory cytokines such as C-reactive protein (CRP), interleukin-6 (IL-6) and tumor necrosis factor-alpha (TNF-alpha) were analyzed. For the study of insulin resistance the homeostasis model assessment (HOMA) index was calculated: HOMA = insulin (mcU/mL) × (glycemia (mmol/l)/22.5) [37].

### 2.4. Statistical Analysis

Patients were classified into four groups: Subjects with no sarcopenia neither admission nor discharge (control group); subjects with sarcopenia at admission and at discharge (chronic sarcopenia group); subjects with no sarcopenia at admission but develop sarcopenia during the hospital stay (incident sarcopenia group) and patients with sarcopenia at admission but revert this state during the hospital stay (reverted sarcopenia group). Variables are presented as the median and inter-quartile range (IQR) with the exception of categorized variables, which are presented as frequencies. Differences between the groups were assessed by the analysis of variance (ANOVA) for continuous data and by Pearson’s χ^2^ test for categorical data. The change of variables such as weight, SMI and hand-grip strength was calculated by the difference between values at discharge minus the values at admission. In order to investigate relevant factors associated with the risk of sarcopenia (chronic and incident) multiple logistic regression analyses were performed. Thus, the relationship between sarcopenia and clinical and functional variables was estimated by deriving odds ratios (ORs) from multiple logistic regression models. Sarcopenia (incident and chronic) was included as the dependent variable, and age, sex, length of hospital stay, functional ability (gait speed, hand-grip strength and Barthel index,), cognitive performance, nutritional assessment and body mass index (BMI) as independent factors.

In order to investigate the influence of sarcopenia (incident, chronic and the combination of the two types), as well as clinical and functional variables on the risk of mortality after 7-years of follow-up survival analyses were performed by means of Cox regression and Kaplan–Meier plots. The duration of the follow-up was calculated as the interval between the date of study entry (admission to the rehabilitation unit) and the date of death, loss to follow-up or the date the follow-up ended (31st of July of 2019), whichever came first. All the variables fitted the proportional hazard assumption. The Cox regression models were adjusted for potential confounders; sex, age and centre.

Values *p* < 0.05 were considered significant. Statistical analysis was done using SPSS and STATA.

## 3. Results

During the study period 206 patients with hip fracture were admitted of whom nine patients were excluded (five due to a serious clinical condition, two due to colon cancer and two due to having periprosthetic fractures). Of the 197 patients eligible for this study, 10 (5.1%) subjects died in the hospital. The final sample included a total of 187 subjects (73.8% women) with a mean age of 85.2 ± 6.3 years. Baseline characteristics are presented in Table 1. At admission sarcopenia was more prevalent in women (91.3%), without age differences between men and women (data not showed). Almost all patients lived at their own home before the fracture (99%), and only two (1%) patients lived in nursing homes.

**Figure 1:**
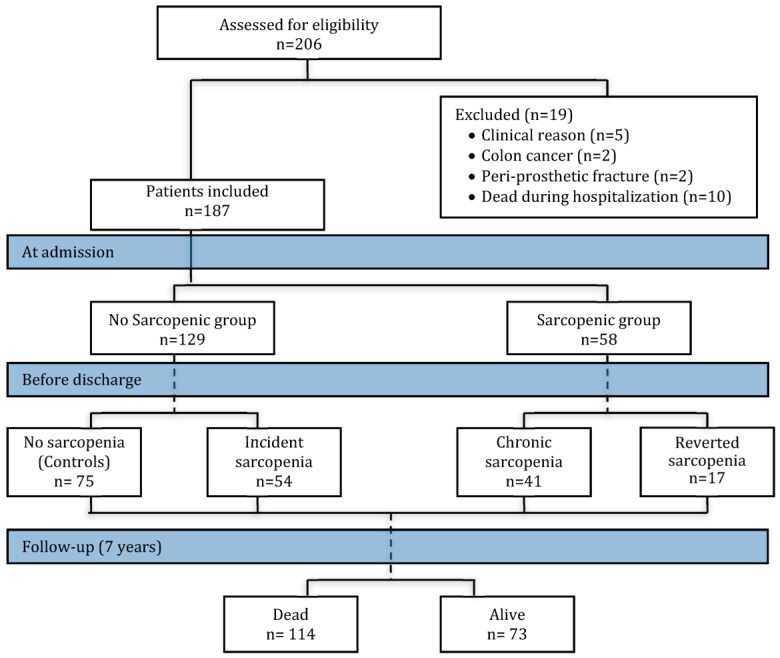
Flow chart of the study population and sarcopenia diagnosis at admission and at discharge. The assessment for sarcopenia was carried out at admission (the sample was divided as the no-sarcopenic and sarcopenic group) and before discharge (no-sarcopenic group at admission was divided as controls and incident sarcopenia, and the sarcopenic group at admission was divided as chronic sarcopenia and reverted sarcopenia). Total mortality was evaluated at 7 years of follow-up. Colored boxes indicate the time period (admission, discharge and follow-up period).

During hospitalization some patients developed sarcopenia and others who had sarcopenia at admission reverted this state. Thus, four different groups of patients were considered in this study: (1) Those patients who were not sarcopenic at admission nor at discharge (controls, *n* = 75), (2) those that were not sarcopenic at admission but developed sarcopenia during their hospital stay (incident sarcopenia, *n* = 54), (3) those with sarcopenia at admission and at discharge (chronic sarcopenia, *n* = 41) and (4) those patients who were sarcopenic at admission, but reverted sarcopenia during the admission period (reverted sarcopenia, *n* = 17; Figure 1, flow chart of study population).

During admission 54 subjects developed sarcopenia, and 17 reverted sarcopenia. Differences between experimental groups were analyzed and are shown in Table 2. The chronic sarcopenia group was older than the other groups. No sarcopenic patients registered higher BMI than the rest of the subjects (*p* < 0.0001). Sex distribution differences were observed between groups (*p* < 0.0001). Men who were sarcopenic at admission reverted this state during the hospital stay. Among the 44 men who were non-sarcopenic at admission, 35 (79.5%) developed sarcopenia during the hospital stay. Regarding women, 53 (91.4%) were sarcopenic at admission and 12 of them reverted sarcopenia (*p* < 0.0001). Only 19 (35.2%) women developed sarcopenia during the hospital stay.

The chronic sarcopenia group registered lower PA value compared to controls (Table 2).

Variables included in the sarcopenia diagnosis criteria (SMI, hand-grip strength and gait speed) were statistically different among groups.

Men registered higher loss of skeletal muscle mass during the hospital stay without differences between experimental groups. On the other hand, women showed significant differences between groups on the change observed in skeletal muscle mass. Women in the incident sarcopenia group showed significantly higher muscle mass loss than the other groups, while those with reverted sarcopenia registered a slightly muscle mass increment reaching significant differences between groups (Table 2).

Functional status as assessed by the Barthel index did not show relevant differences between experimental groups as well as the mini mental state examination. Interestingly, the mini nutritional assessment revealed significant differences between groups (Table 2).

Results from logistic regression analyses are shown in Table 3. Risk factors associated to both incident and chronic sarcopenia were low BMI, low hand-grip strength and low SMI.

In the logistic regression analysis performed considering chronic sarcopenia and reverted sarcopenia groups, main factors associated to sarcopenia reversion were the previous Barthel index (OR 0.95, 95% CI 0.90–0.99), hand-grip strength (OR 0.75, 95% CI 0.57–0.97) and SMI (OR 0.05, 95% CI 0.005–0.63), MNA-SF (OR 0.57, 95% CI 0.34–0.96) and CRP (OR 0.96, 95% CI 0.93–0.99). 

### 3.1. Discharge Differences

At discharge 154 (82.4%) patients returned to their own homes, 19 (10.2%) were institutionalized in nursing homes and 14 patients (7.5%) were derived to another hospital due to complications or to complete the rehabilitation process.

Table 4 shows differences between sarcopenic patients (chronic and incident sarcopenic groups) and no sarcopenic patients (control and reverted sarcopenic groups). At discharge sarcopenia was more prevalent in men that in women (71% vs. 43%, *p* = 0.001). Patients with sarcopenia had lower BMI, higher TNF-alpha, lower hand-grip strength, SMI and gait speed.

### 3.2. Mortality

After a mean follow-up period of 3.9 ± 2.1 years, 114 (61%) patients died (71.9% women vs. 28.1% men, *p* = 0.468) 60.5% were sarcopenic vs. 39.5% non-sarcopenic (*p* = 0.001). Mortality was more frequent among sarcopenic than non-sarcopenic patients (72.6% vs. 48.9%, *p* = 0.001). Deceased were older than alive patients (86.6 ± 6.0 vs. 83.0 ± 6.1 years; *p* = 0.0001).

**Figure 2:**
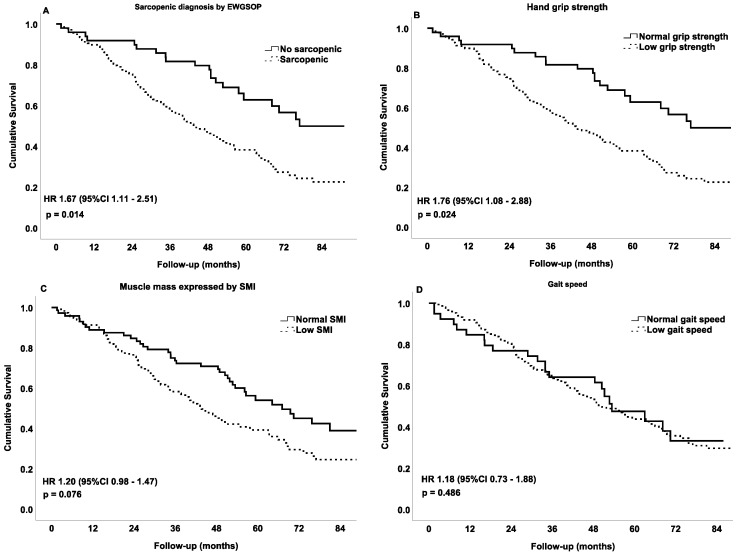
Cox regression of cumulative survival at 7 years of follow-up expressed with the Kaplan–Meier curve (*n* = 187). Variables included at discharge. Models adjusted by age, sex and centre. (**A**) Sarcopenia diagnosis by the revised European Working Group on Sarcopenia in Older People; (**B**) low hand-grip strength <27 kg for men, and <16 kg for women; (**C**) low skeletal muscle index <7.0 kg/m^2^ for men, and <6.0 kg/m^2^ for women and (**D**) low gait speed ≤0.8 m/s.

Cox regression analyses as well as Kaplan–Meier plots showed that sarcopenia was associated with the risk of total mortality (Figure 2, panel A). On the other hand, low hand-grip also presented a significant association with mortality (Figure 2 panel B), while SMI and gait speed showed a non-significant association with the risk of mortality (Figure 2 panel C and D).

When different sarcopenic groups were considered, chronic and incident sarcopenia showed a similar association with the risk of mortality HR: 1.60 (0.93–2.76; *p* = 0.087) and HR: 1.59 (0.97–2.63; *p* = 0.065), respectively, without reaching statistical significance, while the reverted sarcopenia group was not associated to the risk of mortality HR: 0.98 (0.45–2.12; *p* = 0.960).

## 4. Discussion

The present research aimed to identify factors involved in the incidence of sarcopenia, and also to assess the association of sarcopenia with the risk of mortality during a follow-up period of 7 years in older patients with hip fracture.

Our results show that almost 42% of patients developed sarcopenia during hospitalization. As it could be expected, higher muscle mass and hand-grip strength, but also a correct nutritional status, are important protective factors against the incidence of sarcopenia. Many factors favor muscular atrophy, such as age [38], bedrest and a sedentary lifestyle [39]. It is known that the loss of muscle mass is associated with the reduction of strength [40]. However, in line with previous observations [22,41], a significant association between nutritional status and the incidence of sarcopenia was observed in the present study, being patients with higher BMI and MNA-SF those with lower risk for developing sarcopenia. The BMI of the subjects included in this study was within the range defined as normal, but we observed that higher values of BMI (slight overweight) protected against incident sarcopenia. In this sense, it is very important to pay attention to the nutritional status of the elderly with hip fractures and a normal BMI [42,43] since these subjects should be beneficiaries of nutritional supplements, as recommended in the last ESPEN guidelines [44,45]. On the other hand, the relatively high BMI observed in non-sarcopenic subjects could also suggest that being slightly overweight might be a protective factor against adverse events and mortality in older people [16].

Undernutrition is an important mechanism that can promote the onset of sarcopenia [12,46,47], and could explain the increase in the prevalence and incidence of sarcopenia among subjects with no reduced BMI [48]. Insufficient intake contributes to the loss of muscle mass and strength [49], and nutritional supplementation could effectively treat sarcopenia [16,50]. In a previous publication by our group it was described how nutritional supplementation prevents the loss of muscle mass during the functional rehabilitation process after a hip fracture [15].

Considering our results it is interesting to note that 29% of patients reverted sarcopenia during hospitalization being the previous Barthel index, nutritional status, strength and muscle mass the main factors associated to this process.

Sarcopenia [51], as well as frailty [52], are two important reversible geriatric syndromes, and nutritional status has shown to play a very important role on evolution and mortality risk of older adults with hip fracture [16,53,54]. Nutritional intervention [55,56,57], as well as physical exercise [58,59], have been described as effective therapies in the prevention and treatment of sarcopenia.

In the present study only 10% of males were sarcopenic at admission, and the 65% of those who developed sarcopenia during hospitalization were males. In agreement with previous studies [19,60,61], the prevalence of sarcopenia at discharge was greater in males (71%) than in females (43%).

Various possible mechanisms have been proposed to explain the pathophysiological differences of sarcopenia between males and females [62,63]. It will be interesting in the future to analyze in more detail the muscle metabolism of older people after a hip fracture.

In this study we found very high mortality (61%) considering the large follow-up, showing that sarcopenia, defined with the revised EWGSOP criteria, was significantly associated with 1.7 times more risk of death. Other research studies have shown the association between sarcopenia, defined by the EWGSOP, and the increased risk of mortality in older patients admitted to a geriatric acute wards [64].

On the other hand, and as it was expected, hand-grip strength was associated with a higher probability of death with 1.8 times more mortality risk in patients with reduced hand grip strength in comparison to those with normal hand grip strength. Accordingly, previous studies had shown an increase in mortality associated with both low grip strength [65] and loss of hand-grip strength [66] in older community dwelling subjects.

We did not find any studies that evaluated the association of sarcopenia, defined with the revised EWGSOP criteria, and mortality in elderly with hip fractures hospitalized in rehabilitation units.

Regarding gait speed, no significant associations were observed between gait speed and mortality. This result could be explained due to the fact that almost the totality of the sample showed a low gait speed. 

Prior studies have demonstrated that the ability to walk is relatively swiftly recovered following a hip fracture [67], but the recovery of the ability to walk (gait-speed) that patients had before the fracture is usually slow and partial [68], sometimes the recovery is only partial and associated with the onset of a physical disability [69]. In 2011 Studensky et al. already observed the reduced impact of gait speed on survival in very old patients [70].

This study has a number of limitations. Firstly, characteristics of patients not admitted to the rehabilitation units were not available. It is possible that these patients made more rapid progress, and they probably had less sarcopenia, so that the actual prevalence of sarcopenia could be lower in older patients with a hip fracture. Secondly, the measurement of muscle mass was carried out indirectly by BIA, which it is known to present some drawbacks associated with the state of hydration. Nevertheless, we tried to make a measurement of the BIA in the same conditions in all patients. It must be taken into account that BIA is very cheap, easy to use and quickly reproducible, being the better method to assess body composition in ambulatory and bedridden patients. Third, we do not know the cause of death, not knowing whether it was associated directly with sarcopenia or with its possible complications, or if it was secondary to other causes. Finally, taking into account the observational design of the study we cannot discard other possible confounding factors that are not being considered. Nevertheless the sample was very homogeneous and the prospective design of the study allowed us to evaluate the evolution of patients during hospitalization.

Despite these possible limitations, this study has several strengths. The average age of the participants is quite high, so it could be considered as representative of the geriatric population. There are not many studies of sarcopenia, defined according to recently revised EWGSOP criteria, with a very old population and such a large follow-up. Probably the main strength of this study was its pragmatic design [28], which allowed us to hypothesize that the results could be extrapolated to the rest of geriatric patients admitted to rehabilitation units.

## 5. Conclusions and Implications

Sarcopenia is very prevalent in older people with fractures admitted to rehabilitation units and is associated with long-term mortality. Fall prevention and early treatment of sarcopenia (with correction of nutritional deficits and physical exercise) should be two important healthcare policies in the ageing population. Sarcopenia is considered a reversible geriatric syndrome, so future research should assess whether the reversion of sarcopenia is associated with a decrease in mortality. Sarcopenia is associated with malnutrition, which at the same time is associated with adverse events in older people with a hip fracture, so that, an in-depth nutritional assessment to assure the proper treatment may be effective in the prevention or reversion of sarcopenia, improving the recovery of very old patients with a hip fracture.

## Figures and Tables

**Table 1:** Baseline characteristics of the study sample.

	Total *n* = 187
**Age, years**	85.2 ± 6.3
**Sex *n* (%)**	
Female	138 (73.8%)
Male	49 (26.2%)
**BMI kg/m^2^**	25.4 ± 4.6
**Fracture type**	
Intracapsular	89 (47.6%)
Extracapsular	98 (52.4%)
**Type of surgery**	
Replacement	69 (36.9%)
Internal fixation	118 (63.1%)
**Time to surgery**	2 (2–4)
**Non-weight bearing**	29 (15.5%)
**LoS orthopedics (days)**	10 (8–12)
**LoS rehabilitation (days)**	41 (29–57)
**ONS ^&^**	73 (42.6%)
**Previous Barthel index**	85 (60–100)
**MMSE**	22 (16–26)
**MNA-SF ^‡^**	10 (8–12)
**SMI kg/m^2^**	
Female	13.6 ± 2.3
Male	17.2 ± 3.4
**Grip strength kg**	
Female	11.9 ± 5.0
Male	19.6 ± 9.6

MMSE: Mini Mental State Examination, LoS: length of stay, MNA-SF Mini Nutritional Assessment Short Form, ONS: oral nutritional supplementation. ^&^ ONS was available for 184 subjects. ^‡^ MNA-SF was available for 111 patients.

**Table 2:** Admission differences between patients considering different sarcopenic and non-sarcopenic groups.

	No Sarcopenic (Controls) *n* = 75	Incident Sarcopenia *n* = 54	Chronic Sarcopenia *n* = 41	Reverted Sarcopenia *n* = 17	*p*-Value
**Age (year)**	83.9 ± 5.6	86.1 ± 6.8	88.2 ± 4.6 ^‡^	81.1 ± 7.6	<0.0001
**Sex *n* (M/W)**	9/66	35/19	0/41	5/12	<0.0001
**BMI, Kg/m^2^**	28.6 ± 4.7	23.9 ± 3.1 ^‡^	22.2 ± 2.8 ^‡^	23.6 ± 3.0 ^‡^	<0.0001
**Weight, Kg**	70.9 ± 15.0	62.1 ± 10.4 ^‡ &^	53.3 ± 7.4 ^‡ #^	59.0 ± 11.4 ^‡^	0.0001
**SMI, Kg/m^2^**					
Men	7.4 ± 0.4 ^#-$^	5.9 ± 0.6	NA *	6.4 ± 0.3	<0.0001
Women	6.1 ± 0.6 ^&-$^	5.8 ± 0.7 ^&-$^	4.9 ± 0.4	5.2 ± 0.3	<0.0001
**Hand grip, kg**					
Men	28.2 ± 11.3	15.6 ± 6.0 ^‡ $^	NA *	33.7 ± 3.3	<0.0001
Women	13.5 ± 5.9 ^&^	12.3 ± 5.0	9.4 ± 3.0	12.4 ± 2.4	0.0008
**Low hand-grip *n* (%)**	42 (56%)	12 (22.2%)	41 (100%)	17 (100%)	<0.0001
**Gait speed (m/s)**	0.41 ± 0.22	0.36 ± 0.27	0.33 ± 0.22	0.54 ± 0.26 ^&^	0.040
**Phase Angle**	4.5 ± 1.1 ^&^	4.3 ± 1.4	3.7 ± 0.6	4.2 ± 1.1	0.010
**Previous BI**	85 (69–100)	85 (55–100)	80 (60–97)	100 (85–100)	0.090
**Discharge BI**	60 (30–80)	50 (30–75)	55 (25–70)	75 (70–80)	0.069
**MMSE**	22 (16–27)	23 (16–27)	20 (15–25)	23 (17–26)	0.507
**MNA-SF**	11 (10–13)	12 (10–13)	10 (10–11) ^‡^	12 (10–12) ^&^	0.005
**Weight difference ^¥^, kg**	−1.9 ± 3.4	−2.8 ± 3.4	−1.5 ± 2.6	−0.5 ± 3.0	0.054
**SMI difference ^¥^, kg/m^2^**					
Men	−1.4 ± 2.0	−0.8 ± 1.7	NA *	−0.8 ± 1.7	0.872
Women	−0.6 ± 1.2 ^#-$^	−1.8 ± 2.2 ^&-$^	−0.2 ± 0.7 ^$^	0.9 ± 1.2	<0.0001
**Grip strength difference ^¥^, Kg**					
Men	−1.2 ± 5.0	0.4 ± 3.8	NA *	0.5 ± 1.0	0.782
Women	0.96 ± 2.9	−0.33 ± 2.8 ^$^	0.12 ± 2.3 ^$^	3.5 ± 4.8	0.006

Results are expressed as mean ± SD, median (95% CI), or as *n* (%). NA: not available. ^‡^ differences vs. control group; ^#^ difference vs. incident group; ^&^ difference vs. chronic group and $ difference vs. reverted sarcopenia group. * results were not available because no men presented chronic sarcopenia. ^¥^ mean of the difference between values at discharge minus values at admission. BI: Barthel index, BMI: Body mass index, MMSE: Mini Mental State Examination, PA: Phase angle, SMI: Skeletal muscle index. MNA-SF: Mini nutritional assessment-short form.

**Table 3:** Logistic regression analysis considering incident or chronic sarcopenia as dependent variables and clinical indices as independent factors.

Variable	Incident *	*p*	Chronic ^‡^	*p* Value
**BMI**	0.73 (0.64–0.84)	<0.0001	0.64 (0.53–0.76)	<0.0001
**MNA-SF**	0.93 (0.65–1.33)	0.696	0.60 (0.40–0.90)	0.015
**TST ^$^**	0.94 (0.88–1.01)	0.113	0.91 (0.85–0.98)	0.022
**Hand-grip strength**	0.92 (0.85–0.99)	0.038	0.85 (0.77–0.94)	0.002
**SMI**	0.17 (0.07–0.43)	<0.0001	0.002 (0.0002–0.03)	<0.0001
**PA**	0.97 (0.65–1.44)	0.896	0.41 (0.21–0.78)	0.007

Results are expressed as OR (95% CI); TST: Tricipital skinfold thickness. PA: Phase angle. ^$^ TST was available for 49 patients. * Logistic regression analysis adjusted for age, sex and centre. ^‡^ Logistic regression analysis adjusted for age, and centre (not for sex because no male presented chronic sarcopenia).

**Table 4:** Discharge differences in sarcopenic and non-sarcopenic patients.

	Sarcopenia *n* = 95	No Sarcopenia *n* = 92	*p*-Value
**Sex *n* (M/W)**	35/60	14/78	0.001
**Weight, Kg**	56.1 ± 9.4	66.4 ± 13.9	<0.001
**BMI, Kg/m^2^**	22.3 ± 2.8	26.9 ± 4.5	<0.001
**Hb, g/dL**	11.9 ± 1.2	11.6 ± 1.0	0.046
**Total Protein, g/dL**	6.2 ± 0.6	6.2 ± 0.5	0.616
**Albumin, g/dL**	3.5 ± 0.4	3.5 ± 0.4	0.921
**VitD ng/mL**	11 (12.7–24.9)	16 (12–23.5)	0.481
**IL-6, pg/mL**	6.2 (3.6–9.3)	6.0 (3.9–8.4)	0.753
**TNF-alpha, pg/mL**	11.3 (8.0–15.8)	7.5 (5.0–12.4)	0.022
**Barthel index**	50 (30–75)	65 (35–80)	0.053
**MNA-SF**	11.5 (10–12.7)	11.5 (10.5–12.5)	0.880
**PA**	3.9 ± 0.7	4.4 ± 0.8	0.0001
**Hand-grip, kg**	12.8 ± 5.4	16.7 ± 7.9	0.001
Men	17.2 ± 5.6	32.3 ± 4.2	<0.001
Women	10.2 ± 3.3	14.8 ± 5.8	<0.001
**Low grip strength *n* (%)**	95 (100%)	43 (46.7%)	<0.0001
**SMI, Kg/m^2^**			
Men	5.7 ± 0.6	6.9 ± 0.7	<0.001
Women	5.0 ± 0.4	5.9 ± 0.6	<0.001
**Low SMI *n* (%)**	95 (100%)	20 (21.7%)	<0.0001
**Gait speed, m/s**	0.3 ± 0.2	0.4 ± 0.2	0.035
**Low gait speed *n* (%)**	80 (84.2%)	68 (73.9%)	0.083

BMI: Body mass index, Hb: Hemoglobin, IL-6: Interleukin-6, SMI: Skeletal muscle index, TNF-alpha: Tumor necrosis factor-α, VitD: Vitamin D.
